# Letter to the editor by Rosslenbroich, Greiner, Gensorowsky, Grosser, Hasebrook, Schaumburg, Raschke with regard to: Establishment of an interdisciplinary board for bone and joint by Otto-Lambertz et al. https://doi.org/10.1007/s15010-021-01676-9

**DOI:** 10.1007/s15010-022-01849-0

**Published:** 2022-05-13

**Authors:** Steffen B. Rosslenbroich, Wolfgang Greiner, Daniel Gensorowsky, John Grosser, Joachim Hasebrook, Frieder Schaumburg, Michael J. Raschke

**Affiliations:** 1 Department of Trauma, Hand and Reconstructive Surgery, University Hospital Muenster, Muenster, Germany; 2 Institute of Medical Microbiology, University Hospital Muenster, Muenster, Germany; 3 Department for Health Economics and Health Care Management, Bielefeld University, Bielefeld, Germany; 4 Steinbeis University, Berlin, Germany

Dear Editor,

We have read the article by Otto-Lambertz et al. (Establishment of an interdisciplinary board for bone and joint infections) [1] with great interest and we thank the authors for addressing and promoting an interdisciplinary approach in musculoskeletal surgery. The authors promote the implementation of an interdisciplinary team of infectious disease (ID) specialists, orthopedic and trauma surgeons, microbiologists and radiologists to meet the special needs of bone and joint infections. Establishing an interdisciplinary board in modern-day economically driven health systems is challenging since these boards are currently not covered by statutory insurance. We agree with the authors that the increase in quality of care will pay off in the end from an economic perspective. Here, we would like to add certain aspects, which we consider essential for establishing a board for interdisciplinary treatment in musculoskeletal surgery today.

We completely agree that the treatment of osteomyelitis or periprosthetic joint- or fracture-related infections is multi-faceted and complex [1]. Despite existing treatment algorithms, there remains an uncertainty of decision-making in the treatment of bone and joint infection, showing the need for an interdisciplinary standard of care.

Our aging population with predisposing factors like diabetes and vascular comorbidities as well as the rising number of primary arthroplasty procedures [2] and fractures show that our patients are getting more complex and are also in need of an interdisciplinary approach especially treating complications like implant- or fracture-associated infections. The benefit of interdisciplinary teams in the treatment of osteomyelitis has been shown [3], but an infection remains a devastating complication in fracture treatment and primary arthroplasty procedures. Although the incidence of infection in primary arthroplasty is quite low, estimated at 0.3–2%, the incidence for treatment of open fractures is reported to be up to 50%, depending on the degree of soft-tissue damage. The burden on the health system for fractures doubles when comparing the treatment of a closed fracture to an open fracture and even rises sixfold if a fracture-related infection occurs [4, 5]. This rise in costs is mainly explained the prolonged length of stay of these patients in the hospital. We assume big economic and quality potential in optimizing therapy in an interdisciplinary manner and accelerating decision-making by reducing the length of stay in these patients.

Another aspect, which could be of relevance for the health system, is to identify high-risk patients. These patients need to be evaluated and special therapy and prophylaxis strategies have to be developed.

To perform optimized multi-faceted treatment of bone and joint infections as well as prophylaxis in open fractures, we consider additional disciplines an essential part of the interdisciplinary team. In addition to the treatment of the causative pathogens, the role of the soft tissue and blood supply in treatment and prophylaxis is of great importance. We, therefore, believe that involving members of plastic and vascular surgery and angiologists is crucial for any interdisciplinary board in musculoskeletal surgery.

Just like the interdisciplinary team described by Otto-Lambertz et al., our team consists of trauma surgeons, ID specialists, clinical microbiologists and radiologists. From a diagnostic perspective, these additions to the surgical discipline may be sufficient, but concerning decision-making and delivery of treatment in patients with soft-tissue or vascular compromise, the team is incomplete. We, therefore, added plastic- and vascular surgeons as well as members of the angiology department to this interdisciplinary team. Furthermore, pharmacologists and hygienists complete the team.

We think an interdisciplinary board for treatment and prophylaxis needs this composition of disciplines to enable an interdisciplinary approach and to optimize therapy in patients with implant- and fracture-associated infections. Another aspect is that this composition is in the position to develop interdisciplinary treatment algorithms for infections or provide interdisciplinary prophylaxis in musculoskeletal surgery.

Otto-Lambertz et al. focused on the implementation and activities of the interdisciplinary structure. However, outcome parameters such as morbidity, mortality and cost savings were not evaluated. Improvements in these outcomes could positively affect the interdisciplinary approach's effect on treatment quality, clinical outcomes and cost-effectiveness, calling for further investigation.

To answer this question, we were granted public funding to perform a study in which an interdisciplinary team consisting of plastic surgeons, vascular surgeons, angiologists, radiologists, pharmacologists, hygienists, microbiologists and ID specialist will support 33 hospitals in the Northern and Western part of Germany in interdisciplinary decision-making. This team will treat presumably 2.600–3.300 patients with open fractures or surgical complications.

In our opinion, an interdisciplinary structure has the potential to bring main benefits, which we will investigate in our study:

1. Improving the quality of treatment
2. Improvement of effectiveness for the health system including cost-effectiveness
3. Improving job satisfaction and competency development of the treating physicians

When measuring the quality of treatment in trauma surgery, several factors must be considered. The type of injury, the extent of anatomic reconstruction during surgery, soft-tissue management, perioperative management and rehabilitation protocols as well as patient compliance, to mention a few, are factors which have a major impact on the outcome and make evaluation difficult. For our study, we chose four endpoints: the complication rate after initial surgery (non-union, infection, unplanned surgical revision, need for prolonged antibiotic therapy), the number of applied antibiotics, the functionality of the injured limb, and health-related quality of life.

In addition to the evaluation of the clinical outcome, the cost reduction and cost-effectiveness of the new intervention will be evaluated based on claims data provided by three major statutory health insurance funds. The focus will be on utilization of health services and their costs as well as the duration of incapacity for work and health-related quality of life.

Furthermore, a process evaluation will explore the impact on workload, job satisfaction and technology acceptance during the implementation phase and individual onboarding processes of the caretakers as well as a pre–post comparison contrasting treatment and control groups.

We look forward to investigating the potential of interdisciplinary teams as described above. We strongly believe that establishing this structure is a vital measure to optimize complication management and interdisciplinary prophylaxis.
